# Investigating heavy quarkonia binding in an anisotropic-dense quark-gluon plasma with topological defects in the framework of fractional non-relativistic quark model

**DOI:** 10.1038/s41598-024-83328-0

**Published:** 2025-01-13

**Authors:** M. Abu-shady, H. M. Fath-Allah

**Affiliations:** 1 Department of Mathematics and Computer Sciences, Faculty of Science, Menoufia University, Shebin El-kom, Egypt; 2 Higher Institute of Engineering and Technology, Menoufia, Egypt

**Keywords:** Schrödinger equation, Topological defect, Temperature, Anisotropic plasma, Baryonic chemical potential, Generalized fractional derivative Nikorov-Uavorv method, Phenomenology, Theoretical particle physics

## Abstract

The quark-gluon plasma analysis relies on the heavy quark potential, which is influenced by the anisotropic plasma parameter $(\xi ),$ temperature (t), and baryonic chemical potential (μ). Employing the generalized fractional derivative Nikiforov-Uvarov (GFD-NU) method, we solved the topologically-fractional Schrödinger equation. Two scenarios were explored: the classical model (α = β = 1) and the fractional model (α, β < 1). This allowed us to obtain the binding energy of charmonium $(c\bar{c})$ and bottomonium $(b\bar{b})$ in the 1p state. The presence of the topological defect leads to a splitting between the np and nd states. While increasing the temperature reduces the binding energy, increasing the anisotropic parameter has the opposite effect. Compared to the classical model, the fractional model yields lower binding energies. Additionally, the binding energy further decreases with increasing topological defect parameter, and the influence of the baryonic chemical potential is negligible. We also obtained the wave function for the p-state of charmonium and bottomonium. Here, increasing the anisotropic parameter shifts the wave function to higher values. Moreover, the wave function is lower in the fractional model compared to the classical model. Increasing the topological defect parameter again increases the wave function, while the baryonic chemical potential has no discernible effect.

## Introduction

The groundbreaking work of Matsui and Satz^1^ proposed that heavy quarkonium dissociates during quark-gluon plasma (QGP) production. Refs.^2–4^ explore the theoretical and experimental investigation of quarkonia formation or inhibition within QGP media. Additionally, ultrarelativistic heavy-ion collisions (URHIC), as studied in^5,6^, provide avenues for exploring these phenomena. This multifaceted approach highlights the intriguing nature of this research topic from both theoretical and experimental perspectives.

Furthermore, the extended quark sigma model^7–11^ delves into studying hadron properties and phase transitions under extreme conditions. Notably, one of the most remarkable features of QGP formation is the color screening of static chromoelectric fields^12^.

Recently, a system employing the Cornell potential, beyond its Coulombic component, was developed to generate a heavy quark potential at finite temperature^13^. This is achieved by incorporating a dielectric function that captures the effects of a deconfined medium within the potential. This modification allows for calculations within plasma exhibiting limited momentum-space anisotropy, with studies focusing on the real component^14,15^. However, the presence of finite shear viscosity in the quark-gluon plasma (QGP) necessitates considering momentum-space anisotropy^16–18^. These anisotropies, potentially substantial and long-lasting depending on viscosity strength, are particularly relevant at early stages or near plasma boundaries. Their magnitude scales with increasing viscosity, regardless of its coupling strength. Prior investigations of heavy bound-state properties typically focused on vanishing baryonic chemical potential within the thermal medium^19–21^. Non-vanishing chemical potential, however, received less attention in both perturbative and nonperturbative approaches. To address this gap, thermofield dynamics technique^22,23^ was employed in conjunction with a phenomenological potential model^24^. This allowed for studying the color-screening effect at finite temperature and chemical potential, considering both an error-function-type confining force and a color-screened Coulomb-type potential.

Grand Unification Theories (GUTs) predict that the early universe experienced numerous spontaneous symmetry breaks during its cooling phase, accompanied by phase transitions, these transitions, according to Kibble’s mechanism, can create topological defects—concentrated regions of high energy, their nature depends on the GUT’s specific structure and can be point-like (monopoles), linear (cosmic strings), surface-like (domain walls), or combinations thereof^25,26^.

They were created early in the universe’s history. The variety of theories produced from general relativity theory is a very strong incentive to investigate how particles behave on these geometrical structures, despite the lack of any fundamental evidence for their existence. The importance of quantum systems in space–time with linear defect geometry should, therefore, receive special attention, as they are thought to constitute the most significant topological flaw in our universe^25^.

We can list, for instance, the compression of matter during a moving string’s passage and temperature variations in the Cosmic Microwave Background (CMB) as key effects of the hypothetical presence of cosmic strings. A cosmic string’s gravitational field has peculiar characteristics. A quantum particle at rest near a static, straight line with infinite dimensions wouldn’t be drawn to it and wouldn’t experience any local gravitational fields as a result. This indicates that close to a cosmic string, space is flat locally^26^. It is not a worldwide flatness, though; it is local. A cosmic string can have a range of effects on quantum particle dynamics because of its particular shape, including the generation or destruction of the (e + , e-) pair^27^ and the bremsstrahlung process^28^ in the vicinity of a static nucleus. We become interested in the characteristics of hadrons in such spaces under the influence of a central field as a result of these cosmic string-induced fluctuations of quantum observables.

To explore the behavior of heavy quarkonia in such defects, curved space time with conical geometry induced by a cosmic string is examined. The application of fractional calculus in physics has grown, including its use in high energy physics. Ref.^29^ employs the conformable fractional derivative to express a fractional radial SE for extended Cornell potential in N-dimensional space using the extended Nikiforov-Uvarov (ENU) method. This approach aids in describing heavy-quarkonium energy spectra and complex phenomena within the standard model. Ref.^30^ demonstrates the applicability of the fractional NU method to various potentials (oscillator, Woods-Saxon, Hulthen) for solving fractional radial SEs. However, it is recommended to consider the generalized fractional derivative^31^, successfully applied in studying quarkonium^32,33^. In Ref.^32^, the authors studied some properties of quarkonium at zero temperature and thermodynamic properties under the effect of topological defect. In Ref.^33^, the authors studied the masses of single, double, and triple heavy baryons in the hyper-central quark model.

This study investigates the impact of anisotropic plasma and baryonic chemical potential on heavy quarkonia within the framework of a cosmic-string background, which are not considered in recent works. Employing the generalized fractional derivative Nikiforov-Uvarov (GFD-NU) method, we solve the fractional-radial Schrödinger equation (SE). Our analysis yields the binding energy and wave function of charmonium and bottomonium.

The work is organized as follows: The Generalized fractional Nikiforov-Uvarov with second order parametric generalized fractional differential equation is discussed in section “The generalized fractional Nikiforov-Uvarov (NU) method”. In section “Cosmic string space–time of heavy quarkonia”, we find the bound state solutions to the Schrödinger equation for the heavy quark potential in the cosmic-string background. Section “Results and discussion” of the results describes the binding energy and wave function of heavy quarkonia inside cosmic-string geometry. Section “Summary and conclusion” includes conclusions.

## The generalized fractional Nikiforov-Uvarov (NU) method

A novel approach to fractional calculus, termed the Generalized Fractional Derivative (GFD), has been introduced, offering a substantial improvement over traditional definitions such as the Caputo and Riemann–Liouville derivatives. The GFD framework is characterized by its ability to preserve essential mathematical properties, including the derivative of the product and quotient of functions, Rolle’s theorem, and the mean value theorem. These attributes provide a robust foundation for fractional calculus, facilitating a more straightforward and consistent methodology for addressing fractional differential equations, as elaborated in Ref.^31^.

For a function $\uppsi$: (0,$\infty )\to R,$ the generalized fractional derivative of order $0<\alpha \le 1$ of $\uppsi \left(s\right)$ at $>0$ is defined as

1 $${D}^{GFD}\uppsi \left(s\right)=\underset{\varepsilon \to 0}{\text{lim}}\frac{\uppsi \left(s+\frac{\Gamma \left(\beta \right)}{\Gamma \left(\beta -\alpha +1\right)}\varepsilon {s}^{1-\alpha }\right)-\uppsi \left(s\right)}{\varepsilon };\beta >-1, \beta \epsilon { R}^{+}$$

The properties of the generalized fractional derivative are,

2 $${\text{I}}.\quad {D}^{\alpha }\left[ {\uppsi }_{nl}\left(s\right)\right]={k}_{1}{s}^{1-\alpha }{\stackrel{`}{\uppsi }}_{nl}\left(s\right),$$

3 $${\text{II}}.\quad{D}^{\alpha }\left[{D}^{\alpha }\uppsi \left(s\right)\right]={{k}_{1}}^{2}[\left(1-\alpha \right){s}^{1-2 \alpha }{\stackrel{`}{\uppsi }}_{nl}\left(s\right)+{s}^{2-2 \alpha }{{\uppsi }_{nl}}^{{\prime}{\prime}}\left(s\right),$$

where, $${k}_{1}=\frac{\Gamma [\beta ]}{\Gamma [\beta -\alpha +1]}$$, with $$0<\alpha \le 1$$, $$0<\beta \le 1$$.

By using generalized fractional derivative, the parametric generalized fractional Nikiforov-Uvarov (NU) method is introduced. The second-order parametric generalized differential equation is exactly solved in the fractional form as in Ref.^34^

4 $${D}^{\alpha }\left[{D}^{\alpha }\psi \left(s\right)\right]+\frac{\bar{\tau }\left(s\right)}{\sigma \left(s\right)}{D}^{\alpha }\psi \left(s\right)+\frac{\bar{\sigma} (s)}{{\sigma }^{2}} \psi \left(s\right)=0,$$

where $\bar{\sigma} (s)$,$\sigma (s$) and $\bar{\tau }\left(s\right)$ are polynomials of $2\alpha$-th, $2\alpha$-th and $\alpha$-th degree.

To find the suitable solution for Eq. (4) the differentiation of variables, if the transformation is used

5 $$\Psi \left(\text{s}\right)=\Phi \left(\text{s}\right)y\left(\text{s}\right),$$

As in Ref.^35^, Eq. (4) can be written

6 $$\sigma \left(s\right){D}^{\alpha }\left[{D}^{\alpha }y\left(s\right)\right]+\tau \left(\text{s}\right){D}^{\alpha }y\left(\text{s}\right)+\lambda y\left(\text{s}\right)=0$$

where,

7 $$\sigma \left(s\right)=\pi \left(s\right)\frac{\Phi \left(\text{s}\right)}{{D}^{\alpha }\phi \left(\text{s}\right)},$$

where,

8 $$\pi \left(s\right)=\frac{{D}^{\alpha }\sigma \left(s\right)-\bar{\tau }(s)}{2}\pm \sqrt{{\left(\frac{{D}^{\alpha }\sigma \left(s\right)-\bar{\tau }\left(s\right)}{2}\right)}^{2}-\sigma \left(s\right)+K \sigma \left(s\right),}$$

$\pi \left(s\right)$ is the first degree polynomial, The values of $K$ in the square-root of Eq. (8) is possible to determine whether the expression under the square root is square of expression. This is possible if the discrimination is zero.

and

9 $$\lambda =K+{D}^{\alpha }\uppi \left(\text{s}\right),$$

$\lambda$ is a constant and. Replacing *K* into Eq. (8), we define

10 $$\tau \left(s\right)=\bar{\tau }\left(s\right)+2\pi \left(s\right),$$

the derivative of $\tau$ should be negative^36^, since $\rho (s)>0$ and $\sigma (s)$ $>0$ then this is solution. Then, the new eigenvalue equation becomes

11 $$\lambda = {\lambda }_{n}=-n{D}^{\alpha } \tau -\frac{n\left(n-1\right)}{2}{D}^{\alpha }\left[{D}^{\alpha }\sigma \left(s\right)\right].$$

The hypergeometric type equation has a particular solution with degree $\alpha$. Equation (2) has a solution which is the product of two independent part $y\left(s\right)={y}_{n}(s)$ is $n$ degree polynomial which fulfills the form of the hypergeometric equation

12 $${y}_{n}\left(s\right)=\frac{{B}_{n}}{\rho \left(s\right)}({{D}^{\alpha })}^{n}\left({{\sigma \left(s\right)}^{n} \rho }_{n}\left(s\right)\right),$$

where, ${B}_{n}$ is a constant of normalization and $\rho (s)$ is a function of weight that follows the next equation

13 $${D}^{\alpha }\omega \left(s\right)=\frac{\tau \left(s\right)}{\sigma \left(s\right)}\omega \left(s\right),\omega \left(s\right)=\sigma \left(s\right)\rho \left(s\right),$$

14 $${D}^{\alpha }\left[\sigma \left(s\right)\rho \left(s\right)\right]=\tau \left(s\right)\sigma \left(s\right),$$

### Second order parametric generalized differential equation

The following equation is a general form of the Schrödinger equation which can be obtained by transforming it into a second-order parametric generalized differential equation ^37^.

15 $${D}^{\alpha }\left[{D}^{\alpha }\psi \left(s\right)\right]+\frac{{\alpha }_{1}-{\alpha }_{2} {s}^{\alpha }}{ {s}^{\alpha }\left(1-{\alpha }_{3} {s}^{\alpha }\right)}{D}^{\alpha }\psi \left(s\right)+\frac{- {\xi }_{1 }{s}^{2\alpha }+{\xi }_{2}{ s}^{\alpha }-{\xi }_{3}}{{ ({s}^{\alpha }\left(1-{\alpha }_{3} {s}^{\alpha }\right))}^{2}} \psi \left(s\right)=0.$$

16 $$\bar{\tau }\left(s\right)={\alpha }_{1}-{\alpha }_{2} {s}^{\alpha },$$

17 $$\sigma \left(s\right)={s}^{\alpha }\left(1-{\alpha }_{3} {s}^{\alpha }\right),$$

18 $$\check{\sigma} \left(s\right)=- {\xi }_{1 }{s}^{2\alpha }+{\xi }_{2}{ s}^{\alpha }-{\xi }_{3}.$$

Substituting these into Eq. (8), we obtain

19 $$\pi ={\alpha }_{4}+{\alpha }_{5}{s}^{\alpha }\pm \sqrt{({\alpha }_{6}-\text{K }{\alpha }_{3}) {s}^{2\alpha }+({\alpha }_{7}+{\text{K}}){ s}^{\alpha }+{\alpha }_{8}},$$

where,

20 $${\alpha }_{4}= \frac{1}{2}\left(\alpha -{\alpha }_{1}\right),$$

21 $${\alpha }_{5}=\frac{1}{2 }\left({\alpha }_{2}-2{\alpha }_{3}\varkappa \alpha \right),$$

22 $${\alpha }_{6 }={{\alpha }_{5}}^{2}+{\xi }_{1},$$

23 $${\alpha }_{7}=2{\alpha }_{4}{\alpha }_{5}-{\xi }_{2},$$

24 $${\alpha }_{8}={{\alpha }_{4} }^{2}+{\xi }_{3}.$$

In Eq. (19), the function under square root should be the square of a polynomial according to the NU method, so that

25 $$K=-({\alpha }_{7}+2 {\alpha }_{3}{\alpha }_{8})\pm 2\sqrt{{\alpha }_{8}{\alpha }_{9}},$$

where,

26 $${\alpha }_{9}={\alpha }_{3}{\alpha }_{7}+{{\alpha }_{3}}^{2}{\alpha }_{8}+{\alpha }_{6}.$$

In case *K* is negative in the form

27 $$K=-\left({\alpha }_{7}+2 {\alpha }_{3}{\alpha }_{8}\right)-2\sqrt{{\alpha }_{8}{\alpha }_{9}}$$

So that $\pi$ becomes

28 $$\pi ={\alpha }_{4}+{\alpha }_{5}{s}^{\alpha }-[\left(\sqrt{{\alpha }_{9}}+{\alpha }_{3}\sqrt{{\alpha }_{8}}\right){s}^{\alpha }-\sqrt{{\alpha }_{8}}]$$

From Eqs. (10), (16) and (28), we get

29 $$\tau ={\alpha }_{1}+2{\alpha }_{4}-\left({\alpha }_{2}-2{\alpha }_{5}\right){s}^{\alpha }-[\left(\sqrt{{\alpha }_{9}}+{\alpha }_{3}\sqrt{{\alpha }_{8}}\right){s}^{\alpha }-\sqrt{{\alpha }_{8}}]$$

From Eqs. (10) and (26), we get,

30 $${D}^{\alpha } \tau ={k}_{1} [-\alpha \left({\alpha }_{2}-2{\alpha }_{5}\right)-2 \alpha \left(\sqrt{{\alpha }_{9}}+{\alpha }_{3}\sqrt{{\alpha }_{8}}\right)]$$

$$={k}_{1}[-2{ \alpha }^{2}{\alpha }_{3}-2 \alpha \left(\sqrt{{\alpha }_{9}}+{\alpha }_{3}\sqrt{{\alpha }_{8}}\right)]<0$$

From Eqs. (9 and 11), we get the equation of the energy spectrum

31 $$n{k}_{1 }\alpha {\alpha }_{2}-\left(2\text{n }+1\right){k}_{1 }\alpha {\alpha }_{5}+\left(2\text{n}+1\right){k}_{1 }\alpha \left(\sqrt{{\alpha }_{9}}+{\alpha }_{3}\sqrt{{\alpha }_{8}}\right)+n\left(\text{n}-1\right){{k}_{1 }}^{2}{\alpha }^{2}{\alpha }_{3}+{\alpha }_{7}+2{\alpha }_{3}{\alpha }_{8}+2\sqrt{{\alpha }_{8}{\alpha }_{9}}=0$$

If we take $\alpha =1=\beta$ and ${k}_{1}=1$, we get the classical equation of the energy eigenvalue as Ref.^38^

32 $$n{\alpha }_{2}-\left(2\text{n }+1\right){\alpha }_{5}+\left(2\text{n}+1\right)\left(\sqrt{{\alpha }_{9}}+{\alpha }_{3}\sqrt{{\alpha }_{8}}\right)+\text{n }\left(\text{n}-1\right){\alpha }_{3}+{\alpha }_{7}+2{\alpha }_{3}{\alpha }_{8}+2\sqrt{{\alpha }_{8}{\alpha }_{9}}=0.$$

from Eq. (14), we get

33 $$\rho \left(s\right)={s}^{\frac{{\alpha }_{10}-\alpha }{{k}_{1 }}}{(1-{\alpha }_{3} {s}^{\alpha })}^{\frac{{\alpha }_{11}}{\alpha {k}_{1 } {\alpha }_{3}}- \frac{{\alpha }_{10}}{\alpha {k}_{1 } }- \frac{1}{ {k}_{1}}}$$

From Eq. (12), we get

34 $${y}_{n}={{P}_{n}}^{(\frac{{\alpha }_{10}-\alpha }{{k}_{1 }}, \frac{{\alpha }_{11}}{\alpha {k}_{1 } {\alpha }_{3}} - \frac{{\alpha }_{10}}{\alpha {k}_{1 } }- \frac{1}{ {k}_{1 }})}(1- 2 {\alpha }_{3} {s}^{\alpha })$$

where,

35 $${\alpha }_{10}={\alpha }_{1}+2{\alpha }_{4}+2\sqrt{{\alpha }_{8}}$$

36 $${\alpha }_{11}={\alpha }_{2}-2{\alpha }_{5}+2 (\sqrt{{\alpha }_{9}}+{\alpha }_{3}\sqrt{{\alpha }_{8}})$$

From Eq. (8), the generalized solution of the wave function becomes,

37 $$\psi \left(s\right)={s}^{\frac{{\alpha }_{12}}{{k}_{1 }}}{\left(1-{\alpha }_{3} {s}^{\alpha }\right)}^{\frac{-{\alpha }_{13}}{\alpha {k}_{1 } {\alpha }_{3}} - \frac{{\alpha }_{12}}{\alpha {k}_{1 } }}{{P}_{n}}^{\left(\frac{{\alpha }_{10}-\alpha }{{k}_{1 }}, \frac{{\alpha }_{11}}{\alpha {k}_{1 } {\alpha }_{3}} - \frac{{\alpha }_{10}}{\alpha {k}_{1 } }- \frac{1}{ {k}_{1 }}\right)}\left(1- 2 {\alpha }_{3} {s}^{\alpha }\right)$$

where,

${{P}_{n}}^{(\gamma ,\delta )}$ are Jacobi polynomials.

38 $${\alpha }_{12}={\alpha }_{4}+\sqrt{{\alpha }_{8}}$$

39 $${\alpha }_{13}={\alpha }_{5}-(\sqrt{{\alpha }_{9}}+{\alpha }_{3}\sqrt{{\alpha }_{8}})$$

Some problems, in case ${\alpha }_{3}$ = 0.

40 $$\underset{{\alpha }_{3}\to 0}{\text{lim}}{{P}_{n}}^{(\frac{{\alpha }_{10}-\alpha }{{k}_{1 }}, \frac{{\alpha }_{11}}{\alpha {k}_{1 } {\alpha }_{3}} - \frac{{\alpha }_{10}}{\alpha {k}_{1 } }- \frac{1}{ {k}_{1 }})}(1-{\alpha }_{3} {s}^{\alpha })={{L}_{n}}^{\frac{{\alpha }_{10}-\alpha }{{k}_{1 }}}(\frac{{\alpha }_{11}}{\alpha {k}_{1 } }{s}^{\alpha })$$

41 $$\underset{{\alpha }_{3}\to 0}{\text{lim}}{\left(1-{\alpha }_{3} {s}^{\alpha }\right)}^{\frac{-{\alpha }_{13}}{\alpha {k}_{1 } {\alpha }_{3}} - \frac{{\alpha }_{12}}{\alpha {k}_{1 } }}={e}^{\frac{{\alpha }_{13}}{\alpha {k}_{1 } }{s}^{\alpha }}$$

Equation (37), becomes

42 $$\psi \left(s\right)={s}^{\frac{{\alpha }_{12}}{{k}_{1 }}}{e}^{\frac{{\alpha }_{13}}{\alpha {k}_{1 } }{s}^{\alpha }}{{L}_{n}}^{\frac{{\alpha }_{10}-\alpha }{{k}_{1 }}}(\frac{{\alpha }_{11}}{\alpha {k}_{1 } }{s}^{\alpha })$$

where, ${L}_{n}$ being the Laguerre polynomials.

The second solution of Eq. (27) in the following case

43 $$K=-\left({\alpha }_{7}+2 {\alpha }_{3}{\alpha }_{8}\right)+2\sqrt{{\alpha }_{8}{\alpha }_{9}}$$

then, the wave function is,

44 $$\psi \left(s\right)={s}^{\frac{{{\alpha }_{12}}^{*}}{{k}_{1 }}}{\left(1-{\alpha }_{3} {s}^{\alpha }\right)}^{\frac{-{{\alpha }_{13}}^{*}}{\alpha {k}_{1 } {\alpha }_{3}}\frac{{{\alpha }_{12}}^{*}}{\alpha {k}_{1 } }}{{P}_{n}}^{\left(\frac{{{\alpha }_{10}}^{*}-\alpha }{{k}_{1 }}, \frac{{{\alpha }_{11}}^{*}}{\alpha {k}_{1 } {\alpha }_{3}}\frac{{{\alpha }_{10}}^{*}}{\alpha {k}_{1 } } \frac{1}{ {k}_{1 }}\right)\times }\left(1-2 {\alpha }_{3} {s}^{\alpha }\right)$$

The generalized solution of the energy eigenvalue is,

45 $$n {k}_{1}\alpha {\alpha }_{2}-2\text{n }{k}_{1 }\alpha {\alpha }_{5}+(2\text{n}+1){k}_{1 }\alpha (\sqrt{{\alpha }_{9} }-{\alpha }_{3}\sqrt{{\alpha }_{8}})+\text{n}(\text{n}-1){{k}_{1 }}^{2}{\alpha }^{2}{\alpha }_{3}+{\alpha }_{7}+2{\alpha }_{3}{\alpha }_{8}-2\sqrt{{\alpha }_{8}{\alpha }_{9}}+{k}_{1 }\alpha {\alpha }_{5}=0$$

where,

46 $${{\alpha }_{10}}^{*}={\alpha }_{1}+2{\alpha }_{4}-2\sqrt{{\alpha }_{8}}$$

47 $${{\alpha }_{11}}^{*}={\alpha }_{2}-2{\alpha }_{5}+2 (\sqrt{{\alpha }_{9}}-{\alpha }_{3}\sqrt{{\alpha }_{8}})$$

48 $${{\alpha }_{12}}^{*}={\alpha }_{4}-\sqrt{{\alpha }_{8}}$$

49 $${{\alpha }_{13}}^{*}={\alpha }_{5}- (\sqrt{{\alpha }_{9}}-{\alpha }_{3}\sqrt{{\alpha }_{8}})$$

## Cosmic string space–time of heavy quarkonia

The effect of cosmic string background is modeled under the metric with a disclination^39^, we consider a non-relativistic particle of mass M, dipole moment D moving in the background field of cosmic string. The background is described by the space–time metric. The non-relativistic radial (SE) is presented in as Ref.^40^

50 $$\frac{{d}^{2}{\uppsi }_{nl}(r)}{{dr}^{2}}+\left[-\frac{2M}{{\hslash }^{2}}\text{V}\left(\text{r}\right)+\frac{2\text{M}}{{\hslash }^{2}}{E}_{nl}- \frac{\delta }{{r}^{2}}\right]{\uppsi }_{nl}\left(r\right)=0$$

where $\text{M}=\frac{{m}_{q} {m}_{\bar{q}}}{{m }_{q}+{m}_{\bar{q}} }$ is the reduced mass, where ${m}_{q}, {m}_{\bar{q} }$ are the mass of quark and antiquark^41^, $\delta ={l}_{\left(\zeta \right)}\left({l}_{\left(\zeta \right)}+1\right)$, $\zeta <1$ where $\zeta =1-4 J$ is the topological parameter of the cosmic string, ${l}_{\left(\zeta \right)}={m}_{(\zeta )}+n$ and the quantum number for generalized angular orbits is ${l}_{\left(\zeta \right)}.$ It’s not necessarily the case that the generalized quantum numbers ${l}_{\left(\zeta \right)}$ and ${m}_{(\zeta )}$ are integers. ${l}_{\left(\zeta \right)}={m}_{(\zeta )}+n=\frac{m}{\zeta }+n={l}_{1}-\left(1-\frac{1}{\zeta }\right)m$, where ${l}_{1}$ = 0,1,2,…

The potential takes the form as in Ref.^42^ and references therein which takes the following form

51 $$\text{V}\left(\text{r}\right)=\frac{-{ a}_{1} }{r}\left(1+ \mu \text{ r}\right){e}^{- \mu r}+\frac{2 \sigma }{\mu }\left(1-{e}^{- \mu r}\right)-\sigma r {e}^{- \mu r}$$

where, $\mu$ is the anisotropic screening mass and we get ${a}_{1}=0.385$ ^43,43^$\sigma =0.223$. The effective screening mass is defined^44^

52 $$\mu \left(T,\xi \right)={m}_{D}\left[1- \frac{\xi }{8}S\left(l,m\right)\right],$$

in case $\xi =0,$ it becomes $\mu \left(\xi ,T\right)={m}_{D},$ where, ${m}_{D}$ is Debye mass, $\xi$ is an anisotropic parameter and

53 $$S\left(l,m\right)=\frac{6l\left(l+1\right)-2({m}^{2}+2)}{4l\left(l+1\right)-3},$$

and the Debye mass^45,46^ is given

54 $${m}_{D}=g\left(T\right)*T*\sqrt{\frac{{N}_{c}}{3}+\frac{{N}_{f}}{6}+\frac{{N}_{f}}{2{\pi }^{2}}({\frac{u}{3T})}^{2}},$$

where, ${N}_{c}$ is the number of colors, ${N}_{f}$ is the number of flavors, and $g\left(T\right)$ is the coupling constant^47^. In Eq. (51), the potential is adjusted to mimic the Cornell potential at small temperatures. The inclusion of the Coulomb and linear terms ensures an accurate description of quark-antiquark dynamics. At larger distances, the linear term governs confinement, while the Coulomb term dominates at short distances. These adjustments provide a reasonable approximation of quarkonium states.

Then, we write Eq. (45) under $\mu r<1$ as in Ref.^21^, as follows

55 $$\text{V}\left(\text{r}\right)=A{r}^{3}+B {r}^{2}+C r+\frac{D}{r},$$

where,

56 $$A=\frac{-1 }{2}\sigma {\mu }^{2},$$

57 $$B=\frac{-1 }{2} {a}_{1} {\mu }^{3},$$

58 $$C=\frac{1 }{2} {a}_{1} {\mu }^{2}+\sigma$$

59 $$D=-{ a}_{1}.$$

where, the effect of medium appears through $\mu .$ The radial Schrödinger equation in which the interaction potential is the extended Cornell potential defined as in Ref.^48^ and $z={e}^{-\rho r}$ that we get,

60 $$\frac{{d}^{2}R}{d{r}^{2}}+\frac{1 }{z}\frac{dR}{dr}+\frac{1}{{z}^{2}{(1-z)}^{2}}\{-{\xi }_{1}{z}^{2}+{\xi }_{2}z-{\xi }_{3}\}R(z)=0.$$

where,

61 $${\xi }_{1}=-\frac{2\text{M}E}{{\rho }^{2}}+{t}_{1} {\xi }_{2}=-\frac{4\text{M}E}{{\rho }^{2}}+{t}_{2} {\xi }_{3}=-\frac{2\text{M}E}{{\rho }^{2}}+{t}_{3},$$

with,

62 $${t}_{1}=\frac{20\text{M}A}{{\rho }^{5}}+\frac{12\text{M}B}{{\rho }^{4}}+\frac{6\text{M}C}{{\rho }^{3}},$$

63 $${t}_{2}=\frac{10\text{M}A}{{\rho }^{5}}+\frac{6\text{M}B}{{\rho }^{4}}+\frac{2\text{M}C}{{\rho }^{3}}+\frac{2\text{M}D}{\rho },$$

64 $${t}_{3}=\frac{2\text{M}A}{{\rho }^{5}}+\frac{2\text{M}B}{{\rho }^{4}}+\frac{2\text{M}C}{{\rho }^{3}}+\frac{2\text{M}D}{\rho }+\delta ,$$

and we get the generalized fractional radial part of the Schrödinger equation is

65 $${D}^{\alpha }\left[{D}^{\alpha }R\left(z\right)\right]+\frac{1-{z}^{\alpha } }{{z}^{\alpha }\left(1-{z}^{\alpha }\right)}{D}^{\alpha }R\left(z\right)+ \frac{ -{\xi }_{1 }{z}^{2\alpha }+{\xi }_{2}{z}^{\alpha }-{\xi }_{3}}{{\left({z}^{\alpha }\left(1-{z}^{\alpha }\right)\right)}^{2}}R\left(z\right)=0,$$

By using the following parameters,

66 $${\alpha }_{1}=1, {\alpha }_{2}=1, {\alpha }_{3}=1, {\alpha }_{4}=\frac{1}{2}\left({k}_{1 }\alpha -1\right),$$

67 $${\alpha }_{5}= \frac{1}{ 2}\left({1-2k}_{1 }\alpha \right), {\alpha }_{6}=\frac{1}{4} {{(1-2k}_{1 }\alpha )}^{2}-\frac{2\text{M}E}{{\rho }^{2}}+{t}_{1},$$

68 $${\alpha }_{7}=\frac{1}{2}({k}_{1 }\alpha -1){(1-2k}_{1 }\alpha )+\frac{4\text{M}E}{{\rho }^{2}}-{ t}_{2},$$

69 $${\alpha }_{8}=\frac{1}{4}{({k}_{1 }\alpha -1)}^{2}-\frac{2\text{M}E}{{\rho }^{2}}+ {t}_{3},{\alpha }_{9}=\frac{1}{4}{{k}_{1 }}^{2} {\alpha }^{2} +{t}_{1}-{t}_{2}+{t}_{3},$$

70 $${\alpha }_{10}={k}_{1 }\alpha +2\sqrt{ \frac{1}{4}{({k}_{1 }\alpha -1)}^{2} -\frac{2\text{M}E}{{\rho }^{2}}+ {t}_{3}},$$

71 $${\alpha }_{11}= 2{k}_{1 }\alpha +2\left(\sqrt{\frac{1}{4}{{k}_{1 }}^{2} {\alpha }^{2} +{t}_{1}-{t}_{2}+{t}_{3}}+\sqrt{ \frac{1}{4}{\left({k}_{1 }\alpha -1\right)}^{2} -\frac{2\text{M}E}{{\rho }^{2}}+ {t}_{3}}\right),$$

72 $${\alpha }_{12}={\frac{1}{2}(k}_{1 }\alpha -1)+\sqrt{ \frac{1}{4}{\left({k}_{1 }\alpha -1\right)}^{2} -\frac{2\text{M}E}{{\rho }^{2}}+ {t}_{3}},$$

73 $${\alpha }_{13}=\frac{1}{2}\left({1-2k}_{1 }\alpha \right)-\left(\sqrt{\frac{1}{4}{{k}_{1 }}^{2} {\alpha }^{2} +{t}_{1}-{t}_{2}+{t}_{3}}+\sqrt{ \frac{1}{4}{\left({k}_{1 }\alpha -1\right)}^{2} -\frac{2\text{M}E}{{\rho }^{2}}+ {t}_{3}}\right),$$

the generalized fractional of the energy eigenvalue is obtained,

74 $$E=\frac{{\rho }^{2}}{2\text{M}}\left({t}_{3}+\frac{1}{4}{\left({k}_{1} \alpha -1\right)}^{2}\right)- \frac{{\rho }^{2}}{2\text{M}}{\left(\frac{{t}_{1}-{t}_{3}-{\left(\left(n+\frac{1}{2}\right){k}_{1}\alpha +\sqrt{\frac{1}{4}{{k}_{1}}^{2} {\alpha }^{2} +{t}_{1}-{t}_{2}+{t}_{3}}\right)}^{2}}{2\left(\left(n+\frac{1}{2}\right){k}_{1}\alpha +\sqrt{\frac{1}{4}{{k}_{1}}^{2} {\alpha }^{2} +{t}_{1}-{t}_{2}+{t}_{3}}\right)}\right)}^{2}.$$

The generalized fractional of the wave function is,

75 $$\begin{aligned} R\left(z\right) &= N{z}^{\frac{{\frac{1}{2}({k}_{1}}\alpha -1) +\sqrt{\frac{1}{4}{({k}_{1}\alpha -1)}^{2} -\frac{2\text{M}E}{{\rho }^{2}}+ {t}_{3}}}{{k}_{1}}}{ (1+{z}^{\alpha })}^{\frac{\frac{1}{2}\left({k}_{1}\alpha \right)+ (\sqrt{\frac{1}{4}{{k}_{1}}^{2} {\alpha }^{2} +t}}{{k}_{1 }\alpha } }\\ & {{P}_{n}}^{(\frac{-\alpha +{k}_{1}\alpha + 2\sqrt{ \frac{1}{4}{({k}_{1}\alpha -1)}^{2} -\frac{2\text{M}E}{{\rho }^{2}}+ {t}_{3}}}{{k}_{1} \alpha }, \frac{ {k}_{1}\alpha + 2 \sqrt{\frac{1}{4}{{k}_{1}}^{2} {\alpha }^{2}+ t}}{{k}_{1}\alpha }-\frac{1}{{k}_{1 }})}\times \left(1+2{z}^{\alpha }\right).\end{aligned}$$

where, $t={t}_{1}-{t}_{2}+{t}_{3}$, and N is normalization constant.

## Results and discussion

### Special cases

In fractional model, the energy eigenvalue and wave function are Eqs. (74) and (75) and in the classical model, the energy eigenvalue and wave function are Eqs. (76, 77), in case $(\alpha =\beta =1)$ and ${k}_{1}=1$.

76 $$E=\frac{{\rho }^{2}}{2\text{M}}{t}_{3}- \frac{{\rho }^{2}}{2\text{M}}{\left(\frac{{t}_{1}-{t}_{3}-{\left(\left(n+\frac{1}{2}\right)+\sqrt{\frac{1}{4} +{t}_{1}-{t}_{2}+{t}_{3}}\right)}^{2}}{2\left(\left(n+\frac{1}{2}\right)+\sqrt{\frac{1}{4} +{t}_{1}-{t}_{2}+{t}_{3}}\right)}\right)}^{2}$$

77 $$R\left(z\right)=N{z}^{\sqrt{-\frac{2\text{M}E}{{\rho }^{2}}+ {t}_{3}}}{ (1+z)}^{\frac{1}{2}+\sqrt{\frac{1}{4} +t}}{{P}_{n}}^{(2\sqrt{-\frac{2\text{M}E}{{\rho }^{2}}+ {t}_{3}}, 2 \sqrt{\frac{1}{4} + t})}\times (1+2z)$$

where, $t={ t}_{1}-{t}_{2}+{t}_{3}$, and N is normalization constant.

### Binding energy

In this section, we calculated the binding energy where the parameters of binding energy are ${\text{m}}_{\text{q}}, {\text{m}}_{\bar{\text{q}} }$ of charmonium, and bottomonium are taken from Ref.^41^, where the charm quark mass ${m}_{c}=1.4$ (GeV), the bottom quark mass ${m}_{b}=4.7$ (GeV), ${\text{a}}_{1}=0.385$ ^43,43^$\upsigma =0.223$, ${\text{N}}_{\text{c}}=3$, ${\text{N}}_{\text{f}}=2$.

**Fig. 1:**
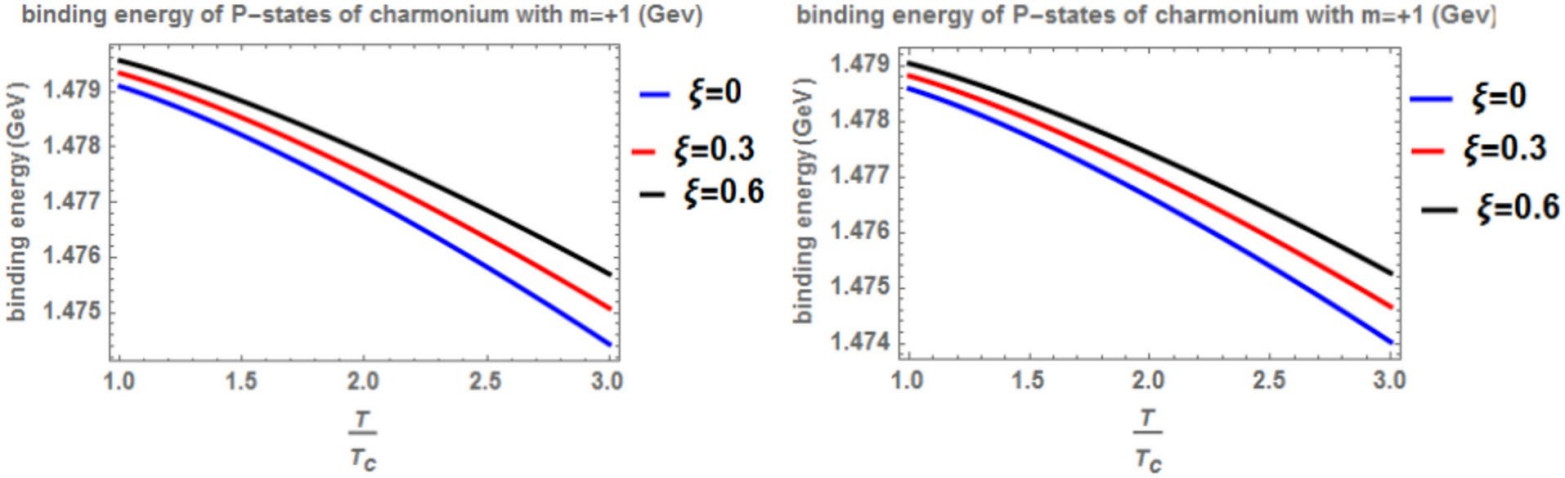
(Left panel) Binding energy with temperature of p-state of charmonium in the classical model ($\alpha =\beta =1)$ with $m=1$ with different values of anisotropic parameter ($\xi )$ and topological defect parameter $\zeta =0.3$. (Right panel) Binding energy with Temperature of p-state of charmonium with $m=1$ in the fractional model ($\alpha =\beta =0.4)$ with $m=1$ with different values of anisotropic parameter ($\xi )$ and topological defect parameter $\zeta =0.3$.

**Fig. 2:**
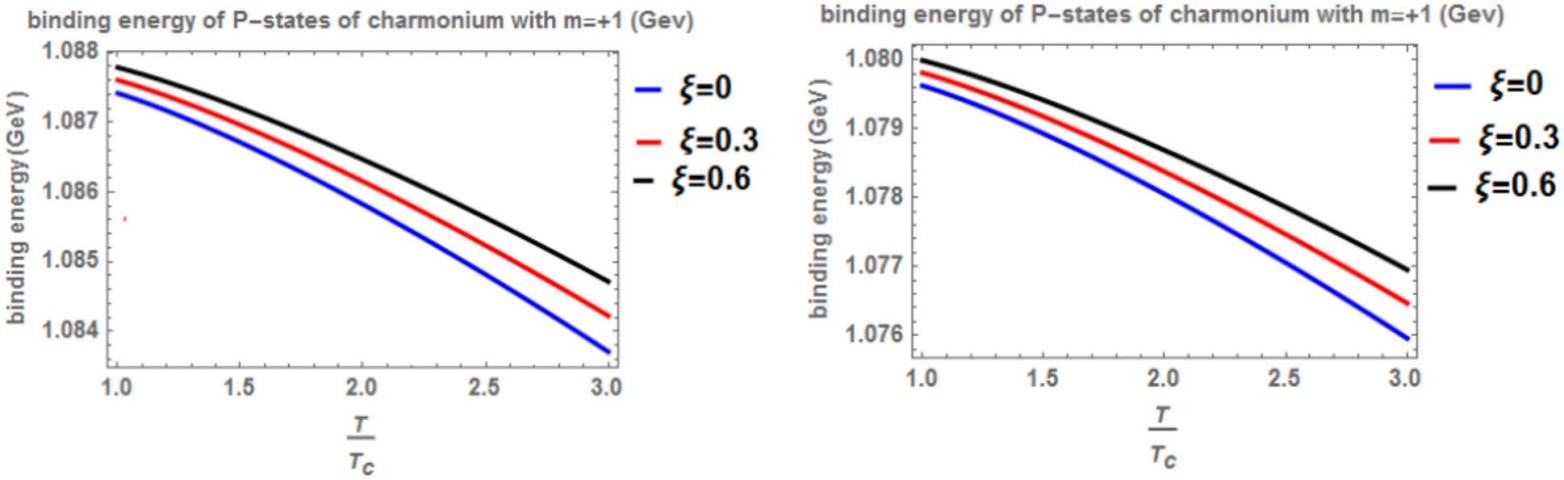
(Left panel) Binding energy with temperature of p-state of charmonium in the classical model ($\alpha =\beta =1)$ with $m=1$ with different values of the anisotropic parameter ($\xi )$ and topological defect parameter $\zeta =0.6.$(Right panel) Binding energy with temperature of p-state of charmonium with $m=1$ in the fractional model ($\alpha =\beta =0.4)$ with $m=1$ with different values of anisotropic parameter ($\xi )$ and topological defect parameter $\zeta =0.6$.

Our investigation of heavy quarkonia (charmonium and bottomonium) within a cosmic-string background begins by determining the heavy quark potential, considering its dependence on radial distance (r), temperature (T), anisotropy parameter $(\xi )$, and chemical potential (μ). Parameters for charmonium and bottomonium are drawn from established literature^41,43^. The fractional parameter in the theory of fractional calculus in quantum mechanics lies within the range 0 < α ≤ 1 and signify the intrinsic behavior of the quantum mechanical systems. The internal behavior of the quantum mechanical system is revealed more at a lower values of the fractional parameter than at α = 1. Analytical solutions of generalized fractional Schrödinger equation For instance, in Figures of binding energy, the energy of the system is lower at a small fractional parameter in comparison with α = 1. This shows that the energy of the system is more bounded at lower fractional parameters than at ordinary quantum mechanics corresponding to α = 1. Next, we employ the generalized fractional derivative Nikiforov-Uvarov (GFD-NU) method to solve the radial Schrödinger equation and obtain the binding energies for 1p states. Two scenarios are explored: “classical model” (α = β = 1) and “fractional model” (α, β < 1). Our primary focus is the impact of the anisotropy parameter ($\xi$) on binding energy. Increasing $\xi$ strengthens the potential due to enhanced effective screening mass, leading to higher binding energy compared to isotropic cases. Conversely, rising temperature weakens the potential and reduces binding energy. We also examine the influence of the topological defect parameter. Its presence induces a splitting between np and nd states, reminiscent of the Zeeman effect where a magnetic field interacts with energy levels. Increasing this parameter strengthens the binding energy. Finally, the effect of the chemical potential is found to be negligible on binding energy. Figures 1 and 2 depict the binding energy of the charmonium p-state as a function of temperature for both the classical model (α = β = 1) and fractional model (α = β = 0.4) with m = 1. They also explore the effects of varying the anisotropic parameter ($\xi )$ and the topological defect parameter ($\zeta$). Consistent with previous studies^4,14,19,49,50^, both figures show a decrease in binding energy as temperature increases, regardless of the model. However, increasing the anisotropic parameter leads to a counterintuitive rise in binding energy, suggesting a strengthening of the potential due to enhanced screening. The influence of the topological defect parameter ($\zeta$) is also visualized, with values of 0.3 and 0.6 considered. While its impact is less pronounced, a slight increase in binding energy is observed with increasing $\zeta$.

We take the baryonic chemical potential parameter ($u$) in this figure in two cases the first case $u=0$, the second case $u=0.3$ and we take these values in all figures and then we observe the inclusion of a chemical potential has little effect on the binding energy. Comparing this figure with Fig. 1, it is observed that increasing the topological defect parameter leads to a decrease in the binding energy, similar to Fig. 2.

**Fig. 3:**
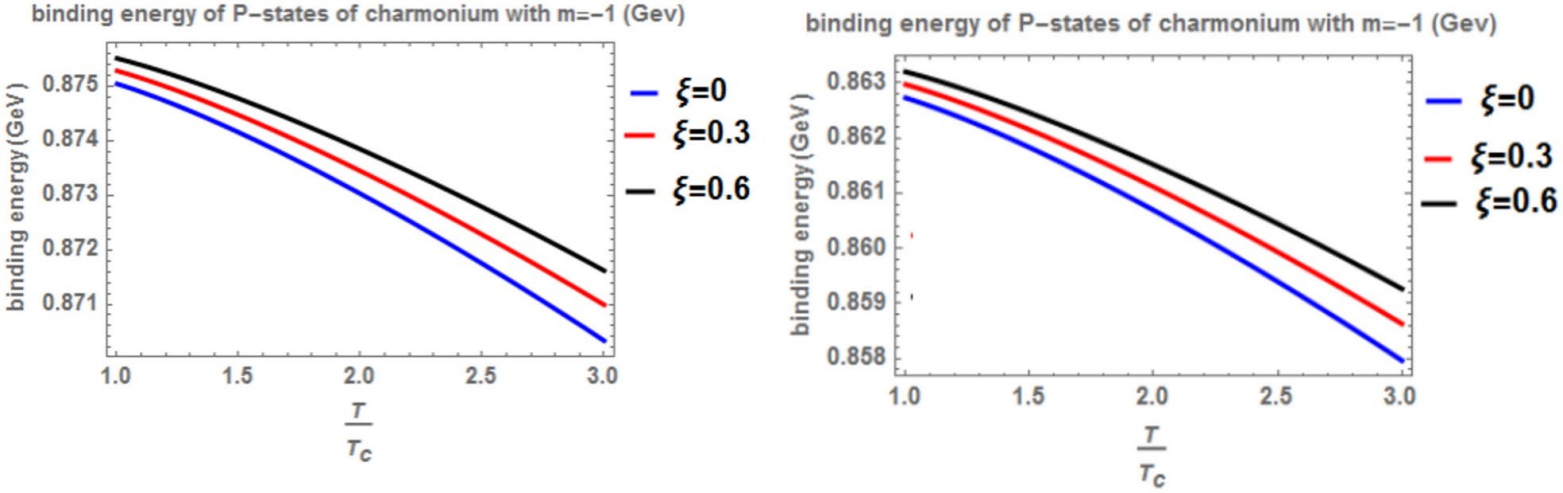
(Left panel) Binding energy with temperature of p-state of charmonium in the classical model ($\alpha =\beta =1)$ with $m=-1$ with different values of the anisotropic parameter ($\xi )$ and topological defect parameter $\zeta =0.3.$(Right panel) Binding energy with temperature of p-state of charmonium with $m=-1$ in the fractional model ($\alpha =\beta =0.4)$ with different values of the anisotropic parameter ($\xi )$ and topological defect parameter $\zeta =0.3$.

**Fig. 4:**
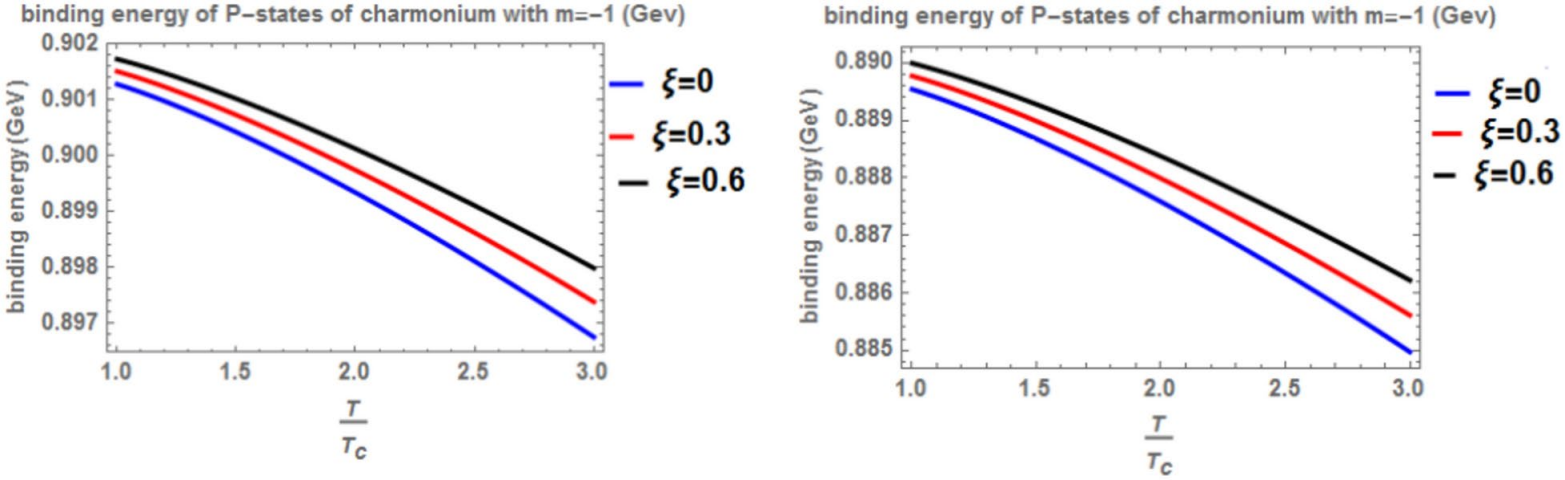
(Left panel) Binding energy with temperature of p-state of charmonium in the classical model ($\alpha =\beta =1)$ with $m=-1$ with different values of isotropic ($\xi )$ and topological defect $\zeta =0.7$. (Right panel) Binding energy with temperature of p-state of charmonium with $m=-1$ in the fractional model ($\alpha =\beta =0.4)$ with $m=-1$ with different values of the anisotropic parameter ($\xi )$ and topological defect parameter $\zeta =0.7$.

**Fig. 5:**
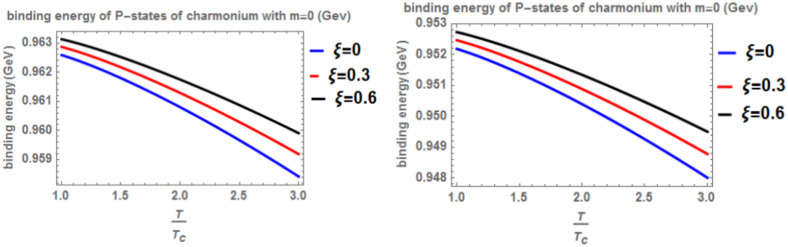
(Left panel) Binding energy with temperature of p-state of charmonium in the classical model ($\alpha =\beta =1)$ with $m=0$ with different values of the anisotropic parameter ($\xi )$ and topological defect parameter $\zeta =0.3$. (Right panel) Binding energy with temperature of p-state of charmonium with $m=0$ in the fractional model ($\alpha =\beta =0.4)$ with different values of anisotropic parameter ($\xi )$ and topological defect parameter $\zeta =0.3$.

**Fig. 6:**
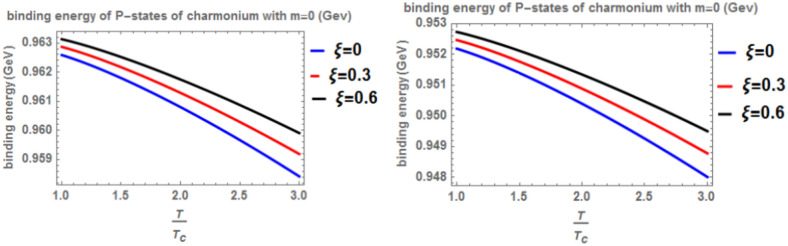
(Left panel) Binding energy with temperature of p-state of charmonium in the classical model ($\alpha =\beta =1)$ with $m=0$ with different values of an isotropic parameter ($\xi )$ and topological defect parameter $\zeta =0.6$. (Right panel) Binding energy with a temperature of p-state of charmonium with $m=0$ in the fractional model ($\alpha =\beta =0.4)$ with different values of the anisotropic parameter ($\xi )$ and topological defect parameter $\zeta =0.6.$

In Fig. 3 presents the binding energy with temperature for the p-state of charmonium, considering the classical model (α = β = 1) and the fractional model (α = β = 0.4) with m = -1. Different values of the anisotropic parameter ($\xi$) and the topological defect parameter $(\zeta =0.3)$ are included. As before, an increase in temperature results in a decrease in binding energy, while an increase in the anisotropic parameter leads to an increase in binding energy. The inclusion of a chemical potential does not have a significant effect on the binding energy. In this case, the binding energy is lower compared to Fig. 1. Lastly, Fig. 4 shows the binding energy with temperature for the p-state of charmonium, considering the classical model (α = β = 1) and the fractional model (α = β = 0.4) with m = -1. Different values of the anisotropic parameter $(\xi )$ and the topological defect parameter $(\zeta =0.7)$ are included. The trends in binding energy with temperature and anisotropic parameter are consistent with previous cases, where an increase in temperature results in a decrease in binding energy, and an increase in the anisotropic parameter leads to an increase in binding energy. No significant effect is observed when including a chemical potential. In this case, the binding energy is higher compared to Fig. 3. In Figs. 5 and 6, when considering the case where m = 0, no effect on the binding energy is observed when using the topological defect parameter. Again, an increase in temperature leads to a decrease in the binding energy, while an increase in the anisotropic parameter increases binding energy. The fractional binding energy also decreases in this case, and including a chemical potential does not affect the binding energy.

**Fig. 7:**
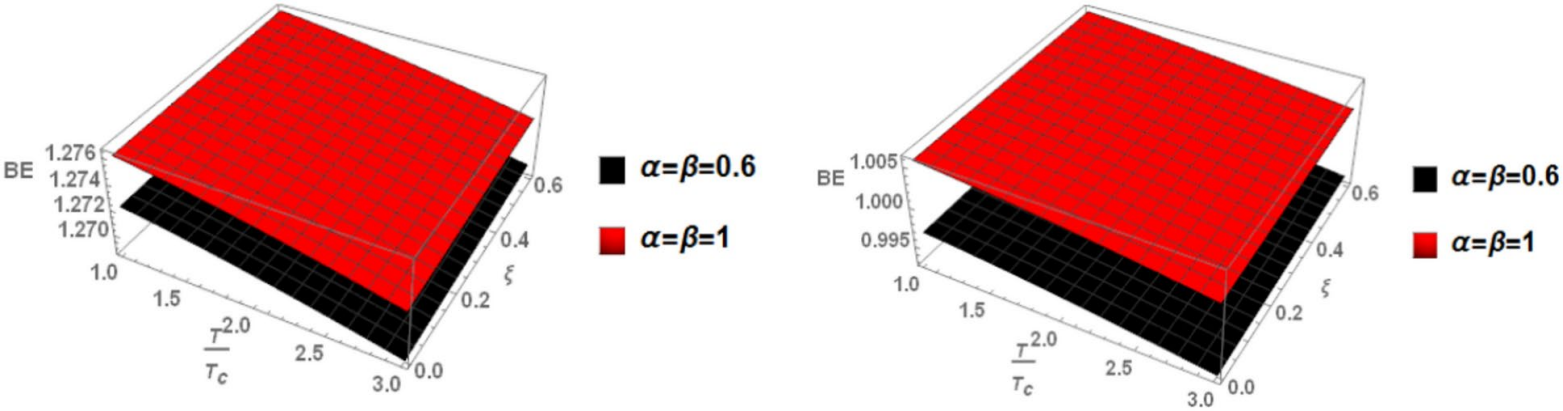
(Left panel) Binding energy with temperature and anisotropic parameter of p-state of charmonium with $m=1$ with different values of $\alpha ,\beta$ and topological defect parameter $\zeta =0.4.$ (Right panel) Binding energy with temperature and anisotropic parameter of p-state of charmonium with $m=1$ with different values of $\alpha ,\beta$, and topological defect parameter $\zeta =0.8.$

Finally, in Fig. 7, we examine the relationship between binding energy, temperature, and anisotropic parameter for the p-state of charmonium with m = 1. Two cases are considered: the classical model and the fractional model, both with a topological defect parameter of $\zeta =0.4$ in the left panel and $\zeta =0.8$ in the right panel.

**Fig. 8:**
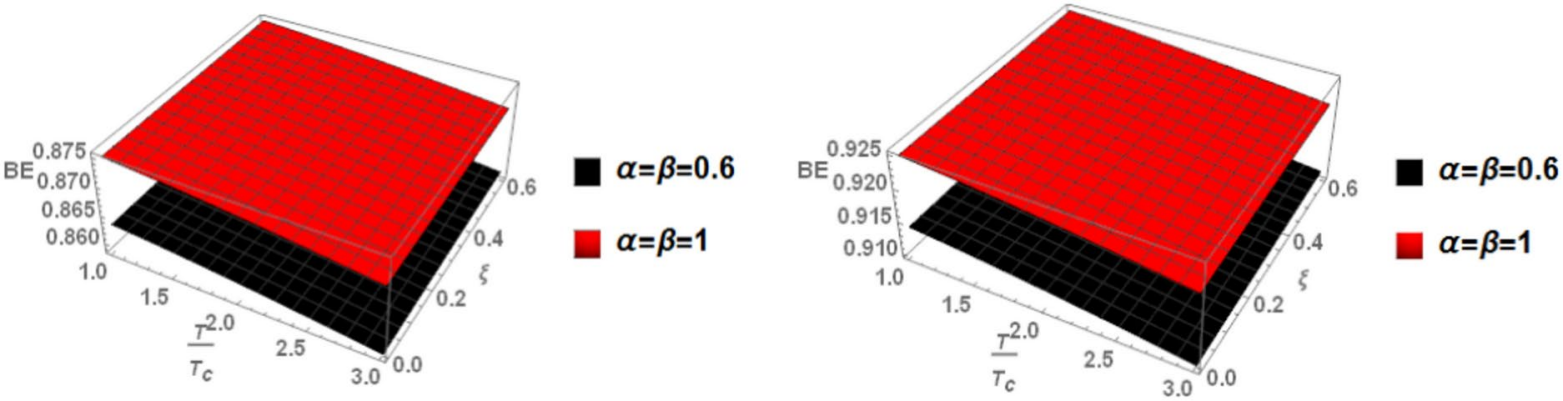
(Left panel) Binding energy with temperature and anisotropic parameter of p-state of charmonium with $m=-1$ with different values of $\alpha ,\beta ,$ and topological defect $\zeta =0.3.$ (Right panel). Binding energy with temperature and anisotropic parameter of p-state of charmonium with $m=-1$ with different values of $\alpha ,\beta ,$ and topological defect parameter $\zeta =0.8.$

**Fig. 9:**
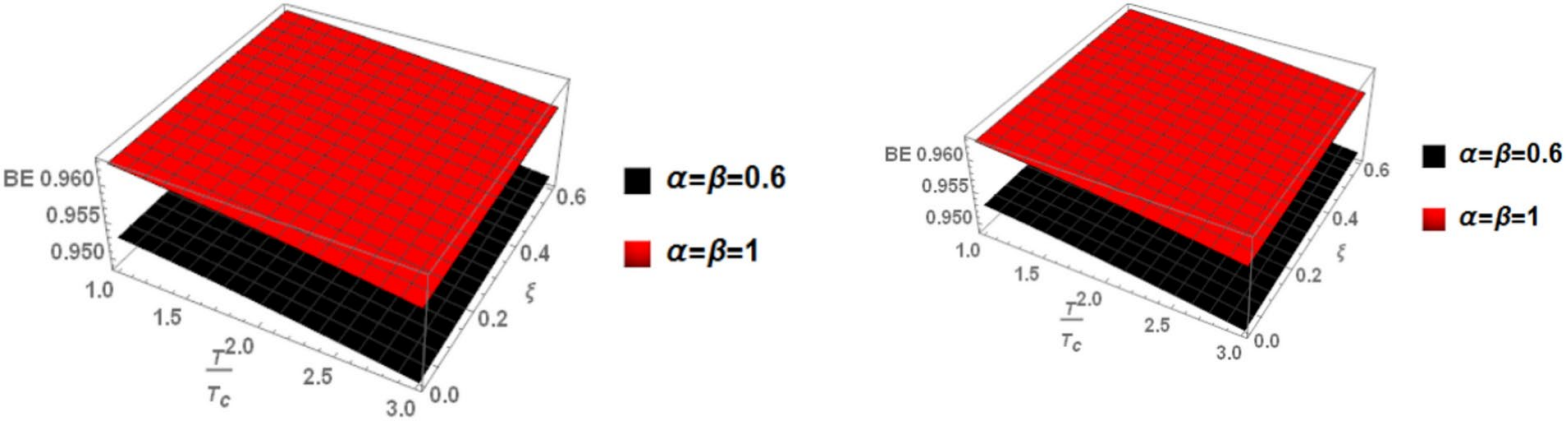
(Left panel) Binding energy with temperature and anisotropic parameter of p-state of charmonium with $m=0$ with different values of $\alpha ,\beta$ and topological defect parameter $\zeta =0.3.$ (Right panel) binding energy with temperature and anisotropic of p-state of charmonium with $m=0$ with different values of $\alpha ,\beta$, and topological defect parameter $\zeta =0.8.$

**Fig. 10:**
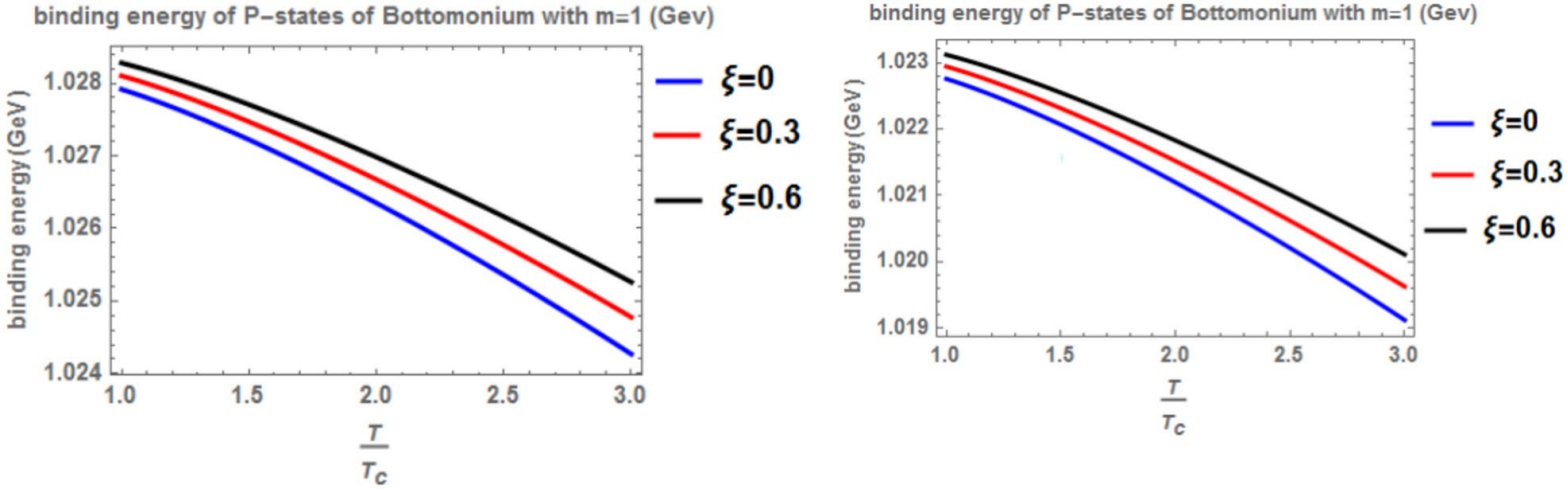
(Left panel) Binding energy with a temperature of p-state of bottomonium in the classical model ($\alpha =\beta =1)$ with $m=1$ with different values of anisotropic parameter ($\xi )$ and topological defect parameter $\zeta =0.3.$ (Right panel) Binding energy with a temperature of p-state of bottomonium with $m=1$ in the fractional model ($\alpha =\beta =0.4)$ with different values of anisotropic parameter ($\xi )$ and topological defect parameter $\zeta =0.3$.

**Fig. 11:**
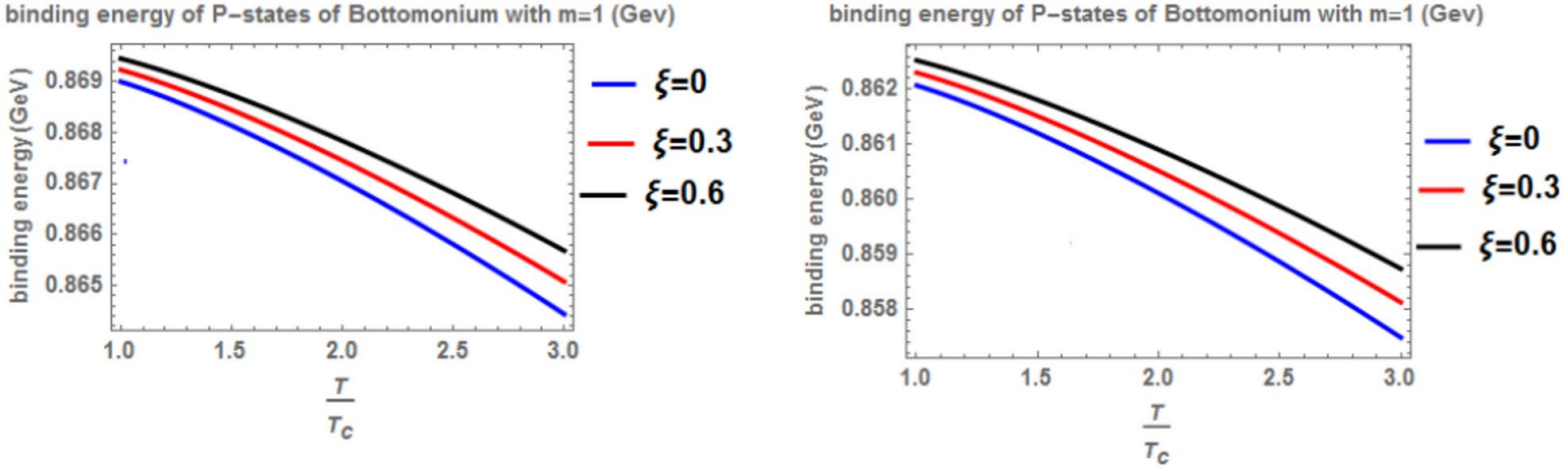
(Left panel) Binding energy with temperature of p-state of bottomonium in the classical model ($\alpha =\beta =1)$ with $m=1$ with different values of the anisotropic parameter ($\xi )$ and topological defect parameter $\zeta =0.6.$ (Right panel) Binding energy with temperature of p-state of bottomonium with $m=1$ in the fractional model ($\alpha =\beta =0.4)$ with different values of anisotropic parameter ($\xi )$ and topological defect parameter $\zeta =0.6$

Figure 8 shows the binding energy with temperature and anisotropic parameter of the p-state of charmonium at m = -1 in two cases: the classical model and the fractional model with topological defect parameters $\zeta =0.4$ (left panel) and $\zeta =0.8$ (right panel). It is observed that as temperature increases, the binding energy decreases while increasing the anisotropic parameter leads to an increase in the binding energy. This observation is consistent with works^4,14,19,49,50^. Furthermore, increasing the topological defect parameter also increases the binding energy, and the binding energy decreases in the fractional model. The baryonic chemical potential does not effect on the binding energy. In Fig. 9, the binding energy with temperature and anisotropic parameter of the p-state of charmonium at m = 0 is plotted. It is found that the topological defect parameter does not affect the binding energy. However, as temperature increases, the binding energy decreases, and with an increase in the anisotropic parameter, the binding energy also increases. Figure 10 presents the binding energy with temperature of the p-state of bottomonium in two cases: the classical model (α = β = 1) and the fractional model (α = β = 0.4) with m = 1 and different values of the anisotropic parameter $(\xi )$ along with a topological defect parameter $\upzeta =0.3.$ It is observed that as temperature increases, the binding energy decreases while increasing the anisotropic parameter leads to an increase in the binding energy. Similarly, in Fig. 11, the binding energy with temperature of the p-state of bottomonium is plotted using the same models as in Fig. 10 but with a different topological defect parameter $\zeta =0.6$. Similar to Fig. 10, it is observed that as temperature increases, the binding energy decreases, while increasing the anisotropic parameter leads to an increase in the binding energy. The binding energy is not affected by the chemical potential. Comparing Fig. 10 and Fig. 11, it is noted that increasing topological defect parameter leads to a decrease in the binding energy, similar to Fig. 11.

**Fig. 12:**
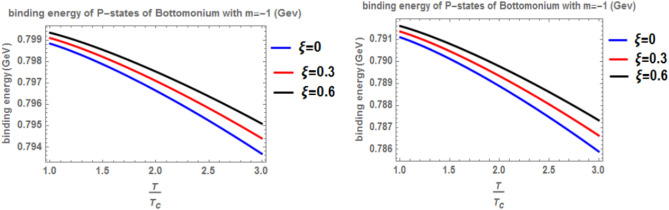
(Left panel) Binding energy with temperature of p-state of bottomonium in the classical model ($\alpha =\beta =1)$ with $m=-1$ with different values of the anisotropic parameter ($\xi )$ and topological defect parameter $\zeta =0.3.$ (Right panel) Binding energy with temperature of p-state of bottomonium with $m=-1$ in the fractional model ($\alpha =\beta =0.4)$ with different values of anisotropic parameter ($\xi )$ and topological defect parameter $\zeta =0.3$

**Fig. 13:**
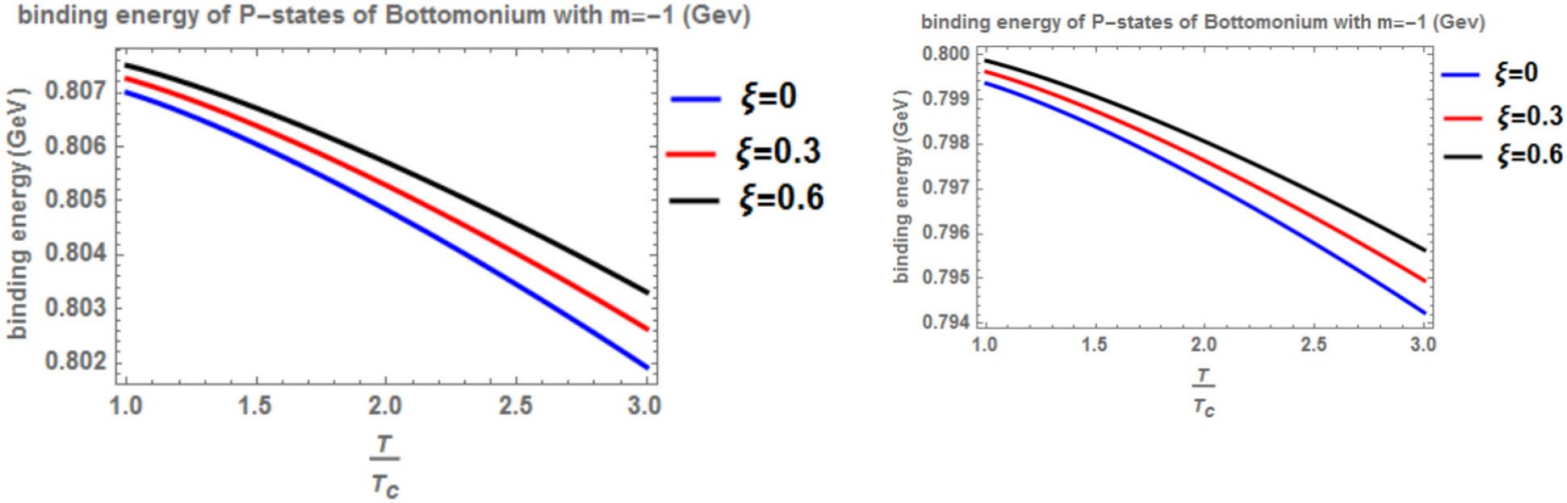
(Left panel) Binding energy with temperature of p-state of bottomonium in the classical model ($\alpha =\beta =1)$ with $m=-1$ with different values of the anisotropic parameter ($\xi )$ and topological defect parameter $\zeta =0.7.$ (Right panel) Binding energy with temperature of p-state of bottomonium with $m=-1$ in the fractional model ($\alpha =\beta =0.4)$ with different values of anisotropic parameter ($\xi )$ and topological defect parameter $\zeta =0.7$

Figure 12 shows the binding energy with temperature of the p-state of bottomonium in two models: the classical model (α = β = 1) and the fractional model (α = β = 0.4) with m = -1 and different values of the anisotropic parameter ($\xi$) along with a topological defect parameter $\zeta$=0.3. Similar to previous models, as temperature increases, the binding energy decreases, while increasing the anisotropic parameter leads to an increase in the binding energy. However, in the case of m = -1, the binding energy is lower compared to Fig. 10. Lastly, Fig. 13 illustrates the binding energy with temperature of the p-state of bottomonium in two models: the classical fractional model (α = β = 1) and the fractional model (α = β = 0.4) with m = -1 and different values of the anisotropic parameter ($\xi$) and topological defect parameter $\zeta$=0.**7.** As in the previous cases, as temperature increases, the binding energy decreases, while increasing the anisotropic parameter leads to an increase in the binding energy.

**Fig. 14:**
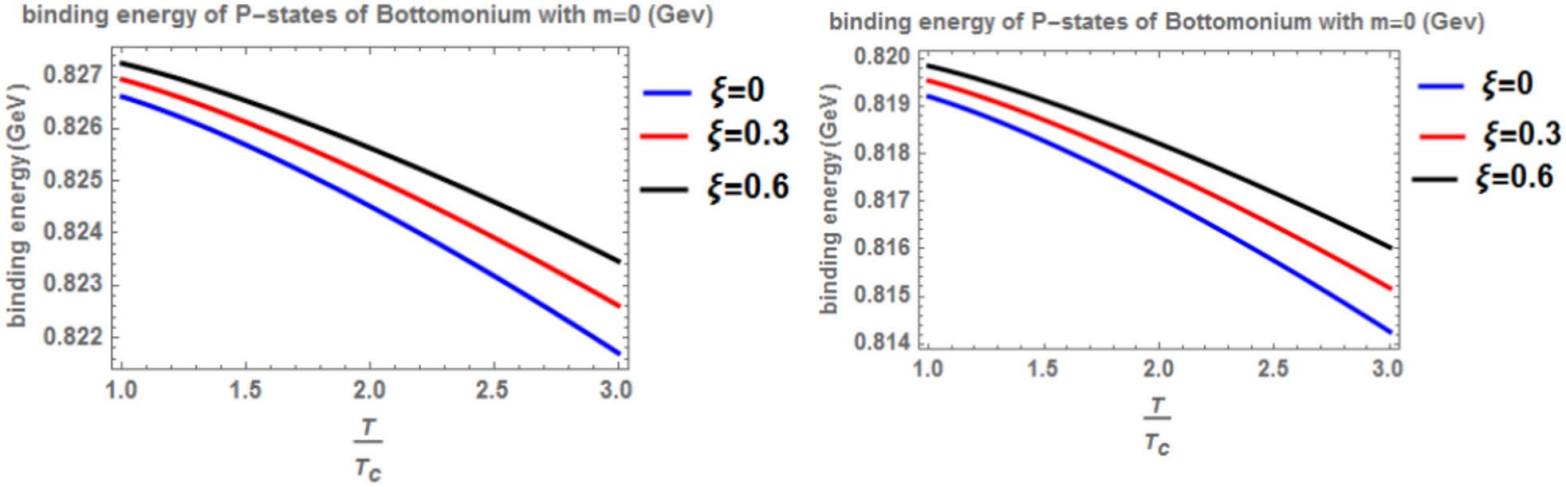
(Left panel) Binding energy with a temperature of p-state of bottomonium in the classical model ($\alpha =\beta =1)$ with $m=0$ with different values of the anisotropic parameter ($\xi )$ and topological defect parameter $\zeta =0.3.$ (Right panel) Binding energy with temperature of p-state of bottomonium with $m=0$ in the fractional model ($\alpha =\beta =0.4)$ with different values of anisotropic parameter ($\xi )$ and topological defect parameter $\zeta =0.3$.

**Fig. 15:**
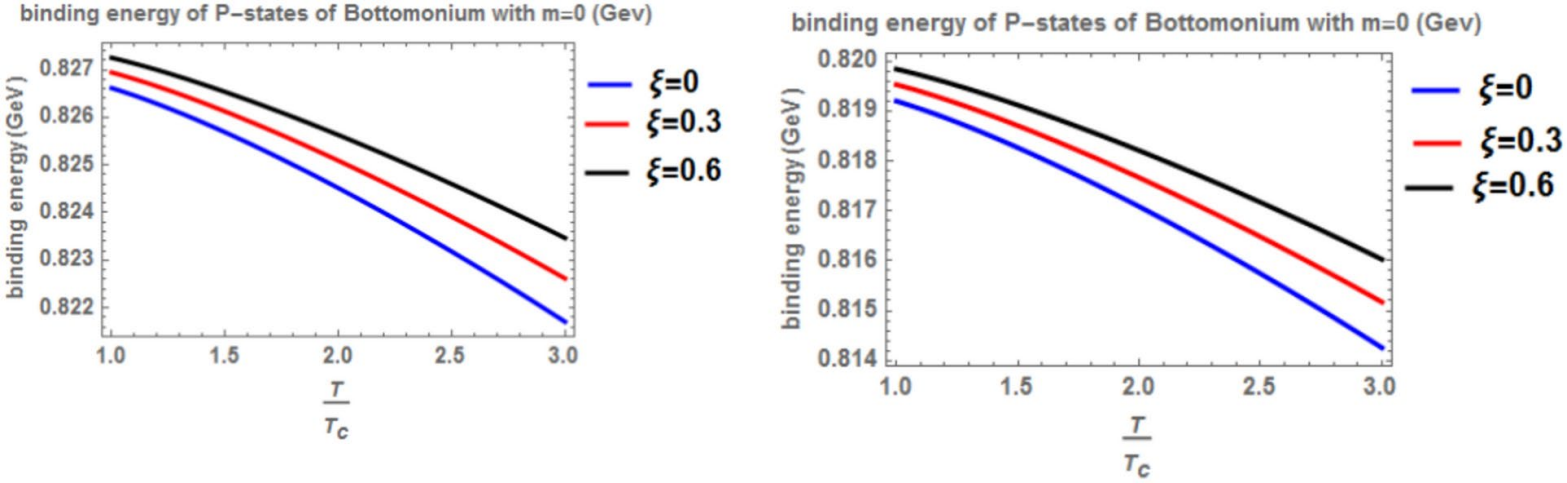
(Left panel) Binding energy with temperature of p-state of bottomonium in the classical model ($\alpha =\beta =1)$ with $m=0$ with different values of the anisotropic parameter ($\xi )$ and topological defect parameter $\zeta =0.6.$ (Right panel) Binding energy with temperature of p-state of bottomonium with $m=0$ in the fractional model ($\alpha =\beta =0.4)$ with $m=1$ with different values of anisotropic parameter ($\xi )$ and topological defect parameter $\zeta =0.6.$

**Fig. 16:**
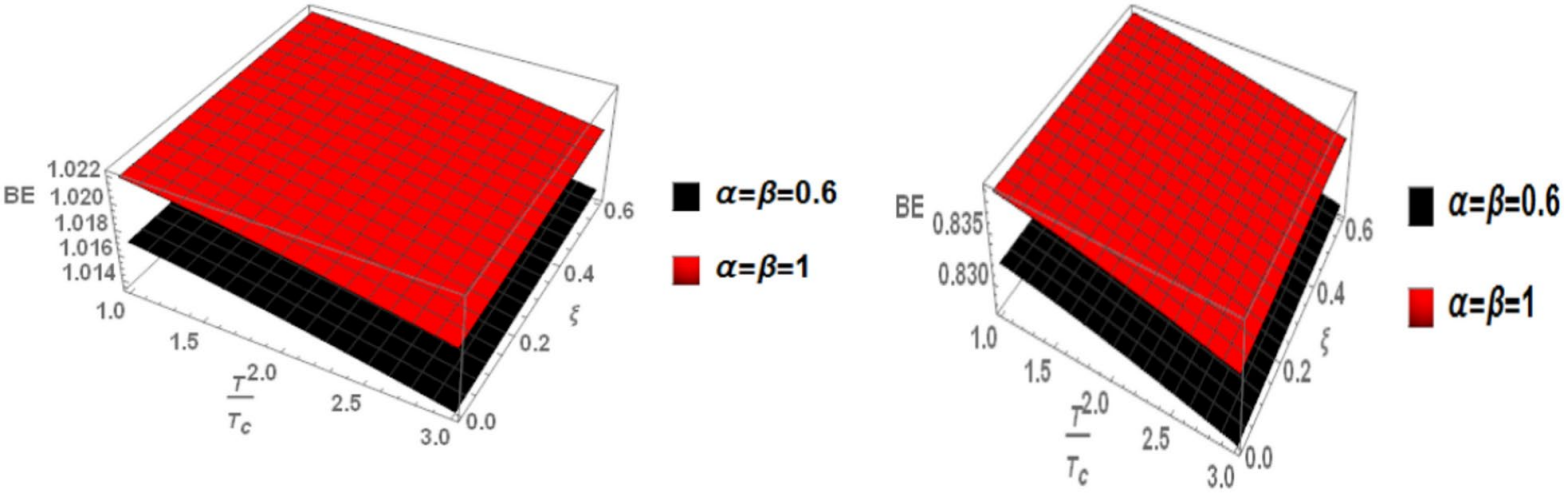
(Left panel) Binding energy with temperature and anisotropic parameter of p-state of bottomonium with $m=1$ with different values of $\alpha ,\beta$, and topological defect parameter $\zeta =0.3.$ (Right panel) Binding energy with temperature and anisotropic parameter of p-state of bottomonium with $m=1$ with different values of $\alpha ,\beta$, and topological defect parameter $\zeta =0.8.$

**Fig. 17:**
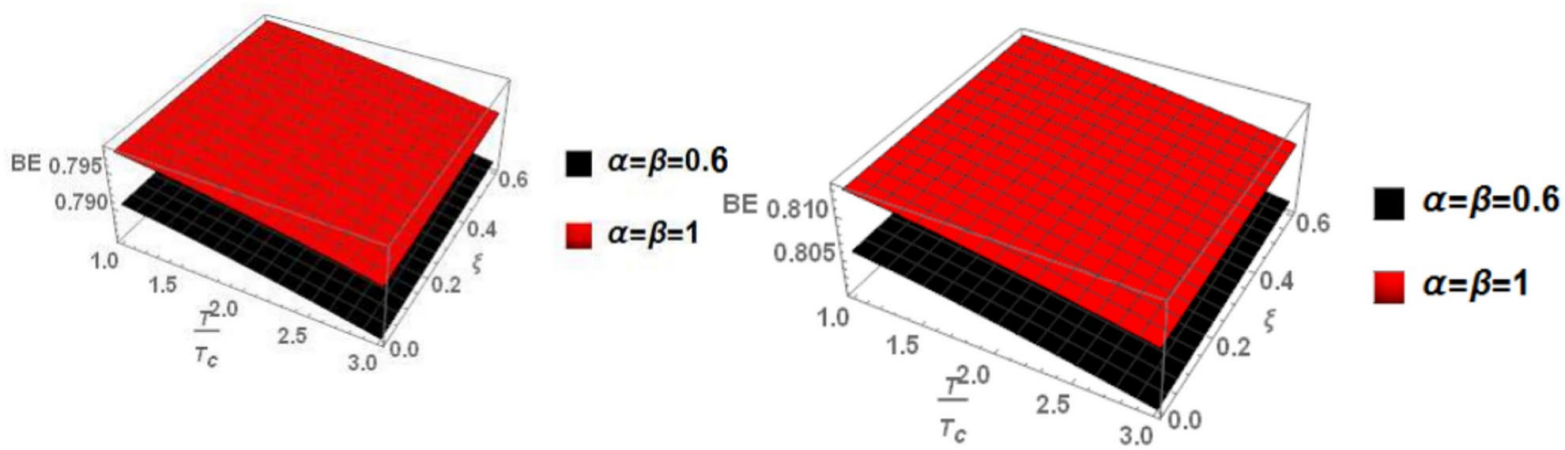
(Left panel) Binding energy with temperature and anisotropic of p-state of bottomonium with $m=-1$ with different values of $\alpha ,\beta$ and topological defect parameter $\zeta =0.3.$ (Right panel) Binding energy with temperature and anisotropic parameter of p-state of bottomonium with $m=-1$ with different values of $\alpha ,\beta$ and topological defect parameter $\zeta =0.8.$

**Fig. 18:**
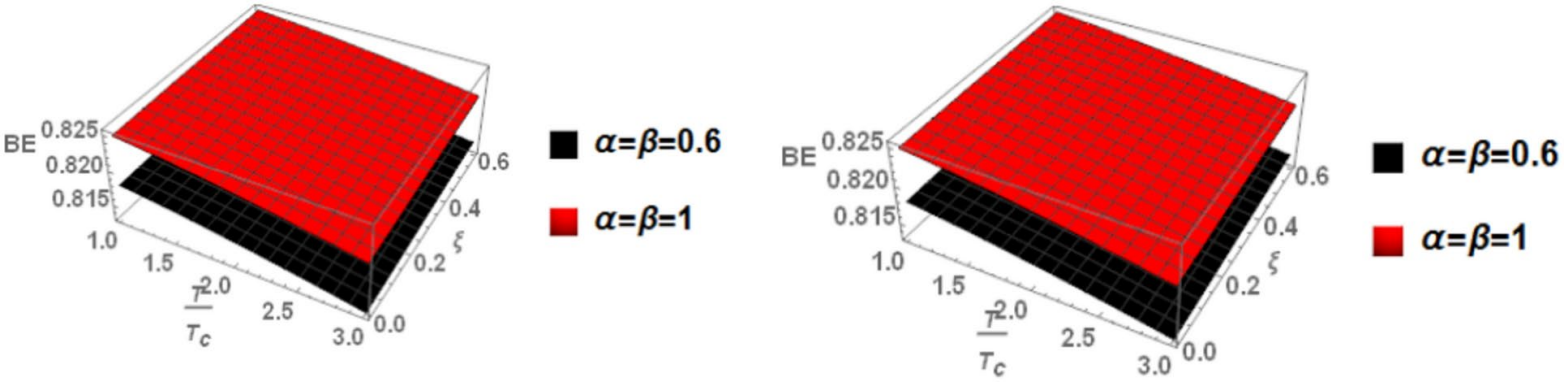
(Left panel) Binding energy with temperature and anisotropic parameter of p-state of bottomonium with $m=0$ with different values of $\alpha ,\beta$ and topological defect parameter $\zeta =0.3.$ (Right panel) Binding energy with temperature and anisotropic of p-state of bottomonium with $m=0$ with different values of $\alpha ,\beta$ and topological defect parameter $\zeta =0.8.$

When the topological defect parameter $(\zeta )$ increases, the binding energy of bottomonium also increases for the m = -1 case, as shown in Fig. 12. This is agreement with Refs.^51,52^. In Ref.^51^, the authors solved the fractional Shro dinger equation. In Ref.^52^, the authors solved the classical Shrodinger equation in an anisotropic parameter in the presence of a topological defect and magnetic flux, they obtained the energy eigenvalues and wave function and they showed that the dissociation energy of bottomonium increase in the presence topological defect, in Figs. 14 and 15 for the m = 0 case, including the topological defect parameter has no effect on the binding energy of bottomonium. We also observe that increasing the temperature leads to a decrease in binding energy, while increasing the anisotropic parameter increases it. The use of the baryonic chemical potential does not affect the binding energy. Figure 16 presents the binding energy of the p-state of bottomonium at m = 1, plotted with varying temperature and anisotropic parameter. Two cases are considered: one in the classical model and the other in the fractional model, with a topological defect parameter of $\zeta$= 0.3 in the left panel and $\zeta$= 0.8 in the right panel. As in all other cases, increasing the temperature leads to a decrease in binding energy, whereas increasing anisotropic parameter increases it. Figure 17 depicts the binding energy of the p-state of bottomonium at m = -1, again with varying temperature and anisotropic parameter in two models: the classical and fractional models. The topological defect parameter remains $\zeta$ = 0.3 in the left panel and $\zeta$= 0.8 in the right panel. Finally, Fig. 18 plots the binding energy of the p-state of bottomonium with temperature and anisotropic parameter for the m = 0 case. While not explicitly mentioned here, the behavior of charmonium is likely similar.

### Radial wave function

**Fig. 19:**
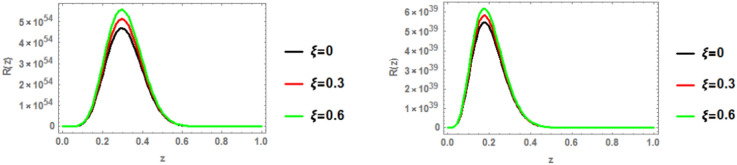
(Left panel) radial wave function of $1p$ of charmonium with z at different values of anisotropic parameter in the classical model ($\alpha =\beta =1)$ at topological defect $\zeta =0.4.$ (Right panel) radial wave function of $1p$ of charmonium with z at different values of anisotropic parameter in the fractional model ($\alpha =\beta =0.7)$ at topological defect $\zeta =0.4.$

**Fig. 20:**
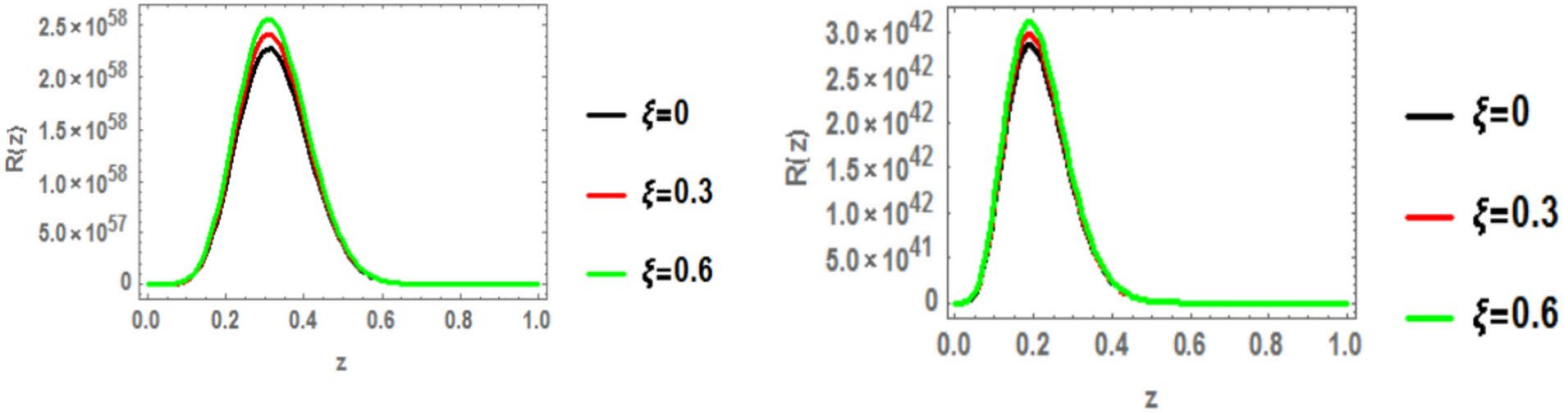
(Left panel) radial wave function of $1p$ of charmonium with z at different values of anisotropic parameter in the classical model ($\alpha =\beta =1)$ at topological defect $\zeta =0.8.$ (Right panel) radial wave function of $1p$ of charmonium with z at different values of anisotropic parameter in the fractional model ($\alpha =\beta =0.7)$ at topological defect $\zeta =0.8.$

**Fig. 21:**
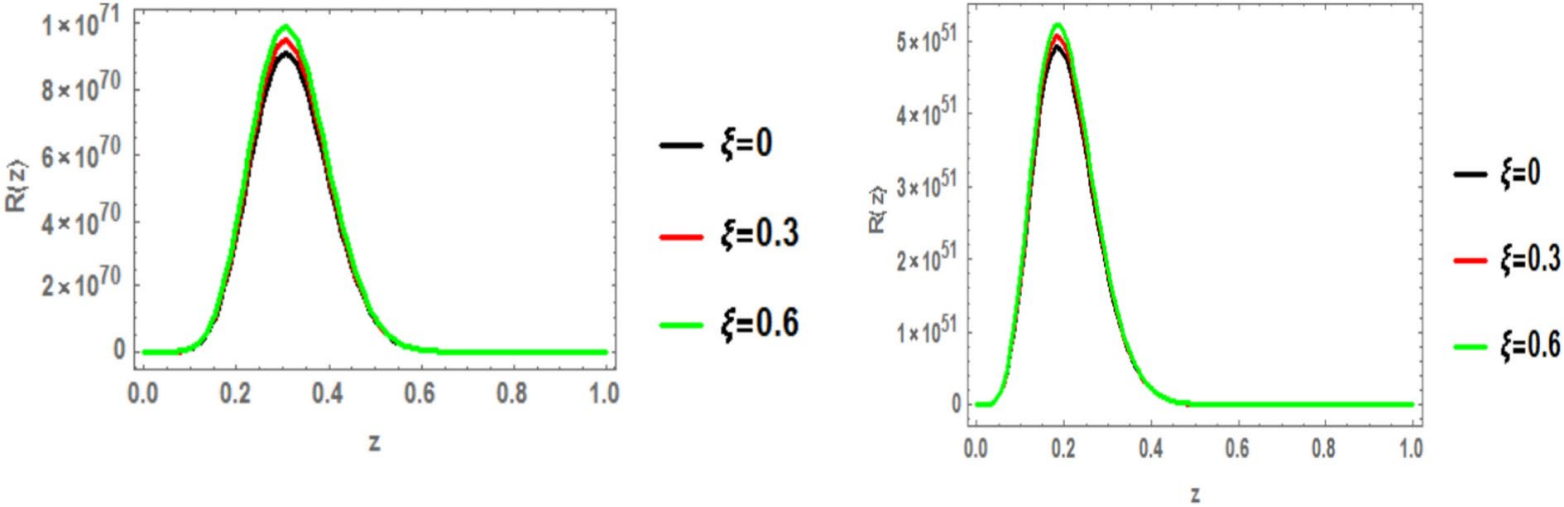
(Left panel) radial wave function of $1p$ of bottomonium with z at different values of anisotropic parameter in the classical model ($\alpha =\beta =1)$ at topological defect $\zeta =0.4.$ (Right panel) radial wave function of $1p$ of bottomonium with z at different values of anisotropic parameter in the fractional model ($\alpha =\beta =0.7)$ at topological defect $\zeta =0.4.$

**Fig. 22:**
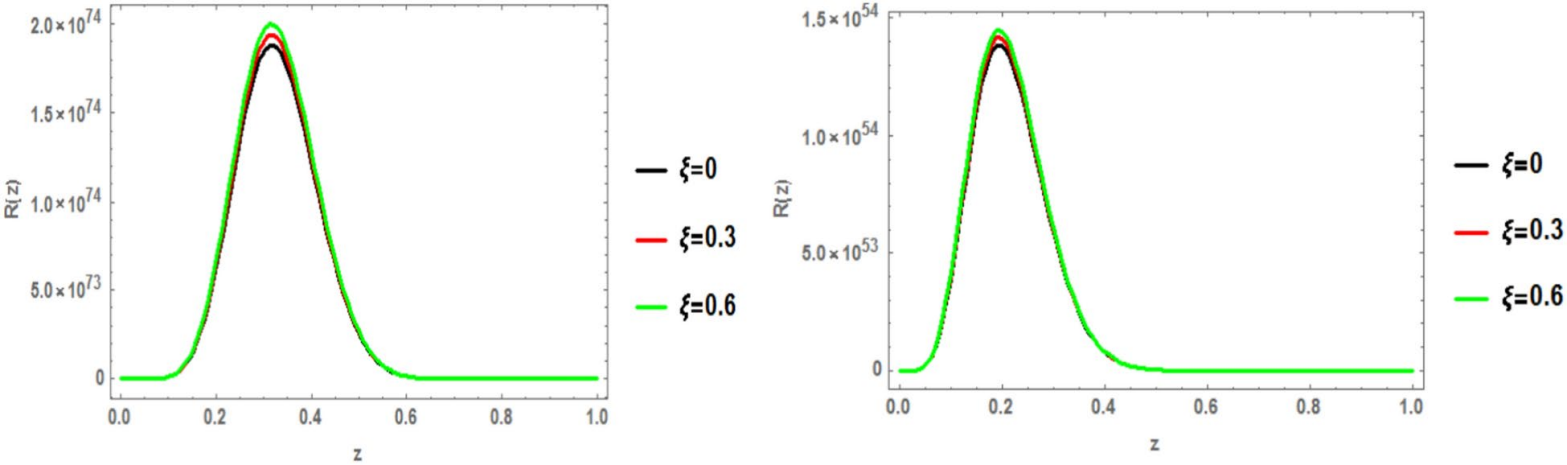
(Left panel) radial wave function of $1p$ of bottomonium with z at different values of anisotropic parameter in the classical model ($\alpha =\beta =1)$ at topological defect $\zeta =0.8.$ (Right panel) radial wave function of $1p$ of bottomonium with z at different values of anisotropic parameter in the fractional model ($\alpha =\beta =0.7)$ at topological defect $\zeta =0.8.$

The radial wave function of the c $\bar{c }$ 1p state is obtained by solving the Schrödinger equation with the heavy quark potential v(r, t, $\xi$, u) using the GFD-NU method. Figure 19 plots the radial wave function at various anisotropic parameter values, considering the influence of the topological defect parameter in both classical and fractional models. The graphs clearly show that the wave function increases with increasing anisotropic parameter. Additionally, the wave function is smaller in the fractional model compared to the classical model. No significant effect of the baryonic chemical potential is observed. Figures 20, 21, and 22 further illustrate that increasing the topological defect parameter leads to an increase in the wave function for both charmonium and bottomonium, regardless of the classical or fractional model.

**Fig. 23:**
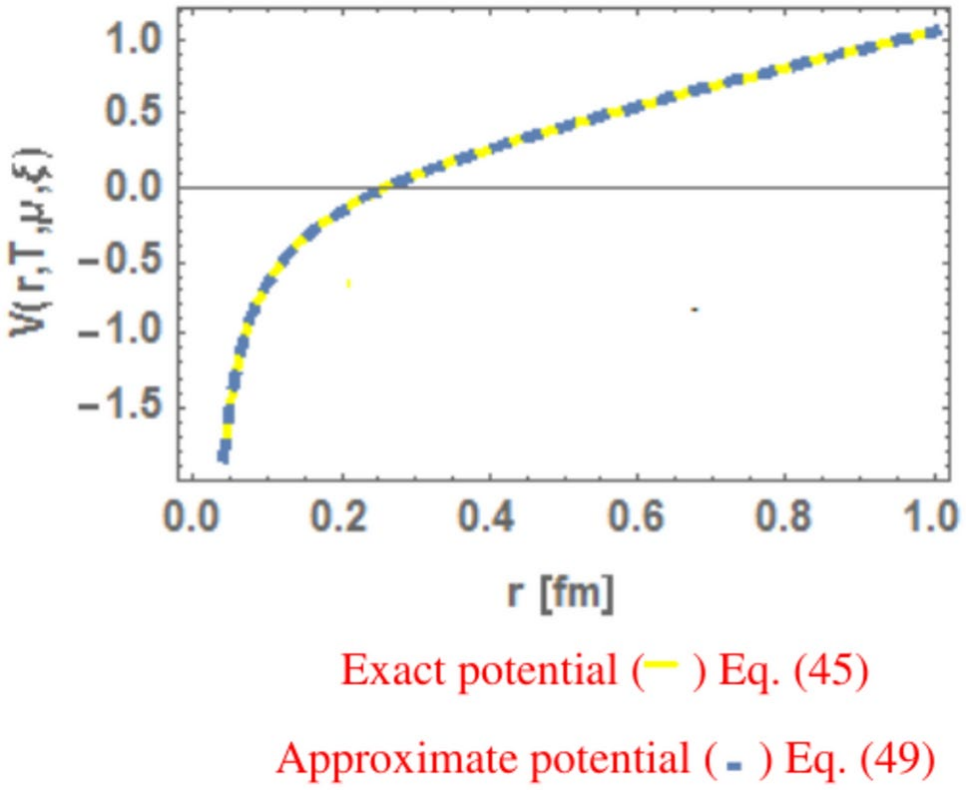
The exact and approximate (potentials are plotted as a function of distance r at T = 0.17 GeV, $u=0.3\text{ GeV}$ and $\zeta =0.1$.

In Fig. 23, the exact potential is plotted of Eq. (45) while the approximate potential is plotted of Eq. (49) to show the two potentials have same behaviors highlighting the contribution of the linear and Coulomb parts. At large distances, the linear part governs quark confinement, while the Coulomb part dominates at short distances. Employing both limits (or limitations) reveals medium modifications induced by anisotropy, temperature, and baryonic chemical potential, consistent with Ref.^21^ when $\mu r<1.$

## Summary and conclusion

In this study, we solved the radial Schrödinger equation by considering a heavy quark potential that varies with temperature, anisotropic parameter, baryonic chemical potential, and the presence of a topological defect. Two models were explored using the GFD-NU method: the classical model (with α = β = 1) and the fractional model (with α, β < 1). The binding energy of charmonium $(c\bar{c})$ and bottomonium $(b\bar{b})$ in the 1p state was calculated in the fractional model, in which we obtained the classical model as a special case at α = β = 1.

It was observed that the splitting between the np and nd states was caused by the topological defect, with the states being split into 2l + 1 components. This suggests that the topological defect’s field interacts with the energy levels similarly to the Zeeman effect induced by a magnetic field. To analyze the influence of the topological defect, the binding energy of the 1p state was plotted for different values of the anisotropic parameter (under the influence of the topological defect) at m = 1,-1,0. The results showed that the binding energy decreased with increasing temperature but increased with increasing anisotropic parameter, which is consistent with previous Refs.^4,14,19,49,50^. Additionally, the binding energy was lower in the fractional model compared to the classical model. In the fractional calculus and its application in quantum mechanics, the parameter α encapsulates the spacetime’s fractal-like properties and modifies the Schrödinger equation’s solutions. It is physically interpreted as a measure of the underlying anisotropic and fractional dynamics, influencing both the binding energy and wave functions. While α does not correspond to a directly measurable quantity, its variation provides insight into how quantum systems deviate from classical behavior under such conditions. Moreover, the effect of the topological parameter on the binding energy at m = 1 was found to be a decrease, while at m = -1 it was an increase this is an agreement with Refs.^51,52^. The topological parameter did not affect the binding energy at m = 0**.** Furthermore, the binding energy is not sensitive to the baryonic chemical potential in either the classical or fractional models.

In addition, we investigated the radial wave function of the p-state of charmonium and bottomonium and examined the effect of the topological defect parameter on the wave function for different values of the anisotropic parameter. It was observed that the wave function increased with increasing anisotropic and topological defect parameters in both the classical and fractional models, with the fractional model yielding lower values compared to the classical model. We plan to extend this work to relativistic quark models, as discussed previously as Refs.^53–56^ with imaginary potential with including the LS and the tensor interactions.

## Data Availability

Data is provided within the manuscript.
